# Moderate Aerobic Training Improves Cardiorespiratory Parameters in Elastase-Induced Emphysema

**DOI:** 10.3389/fphys.2016.00329

**Published:** 2016-08-03

**Authors:** Isabela Henriques, Miquéias Lopes-Pacheco, Gisele A. Padilha, Patrícia S. Marques, Raquel F. Magalhães, Mariana A. Antunes, Marcelo M. Morales, Nazareth N. Rocha, Pedro L. Silva, Débora G. Xisto, Patricia R. M. Rocco

**Affiliations:** ^1^Laboratory of Pulmonary Investigation, Carlos Chagas Filho Institute of Biophysics, Federal University of Rio de JaneiroRio de Janeiro, Brazil; ^2^Laboratory of Cellular and Molecular Physiology, Carlos Chagas Filho Institute of Biophysics, Federal University of Rio de JaneiroRio de Janeiro, Brazil; ^3^Department of Physiology, Fluminense Federal UniversityNiterói, Brazil

**Keywords:** emphysema, remodeling, inflammation, exercise, lung mechanics

## Abstract

**Aim:** We investigated the therapeutic effects of aerobic training on lung mechanics, inflammation, morphometry and biological markers associated with inflammation, and endothelial cell damage, as well as cardiac function in a model of elastase-induced emphysema.

**Methods:** Eighty-four BALB/c mice were randomly allocated to receive saline (control, C) or 0.1 IU porcine pancreatic elastase (emphysema, ELA) intratracheally once weekly for 4 weeks. After the end of administration period, once cardiorespiratory impairment associated with emphysema was confirmed, each group was further randomized into sedentary (S) and trained (T) subgroups. Trained mice ran on a motorized treadmill, at moderate intensity, 30 min/day, 3 times/week for 4 weeks.

**Results:** Four weeks after the first instillation, ELA animals, compared to C, showed: (1) reduced static lung elastance (Est,L) and levels of vascular endothelial growth factor (VEGF) in lung tissue, (2) increased elastic and collagen fiber content, dynamic elastance (E, *in vitro*), alveolar hyperinflation, and levels of interleukin-1β and tumor necrosis factor (TNF)-α, and (3) increased right ventricular diastolic area (RVA). Four weeks after aerobic training, ELA-T group, compared to ELA-S, was associated with reduced lung hyperinflation, elastic and collagen fiber content, TNF-α levels, and RVA, as well as increased Est,L, E, and levels of VEGF.

**Conclusion:** Four weeks of regular and moderate intensity aerobic training modulated lung inflammation and remodeling, thus improving pulmonary function, and reduced RVA and pulmonary arterial hypertension in this animal model of elastase-induced emphysema.

## Introduction

Chronic obstructive pulmonary disease (COPD) is a growing public health issue associated with high morbidity and mortality worldwide (Rubi et al., 2010). COPD is characterized by small-airway disease and parenchymal destruction, resulting in airflow limitation that is not fully reversible (Papaioannou et al., 2010; Yamamoto et al., 2010; Decramer et al., 2015).

Although aerobic training has shown beneficial effects on dyspnea and exercise tolerance in patients with COPD (Amin et al., 2014; Jacome and Marques, 2014), its impact on cardiorespiratory impairment have shown conflicting results (Oliveira et al., 2014). Additionally, experimental studies have reported controversies regarding the effects of aerobic exercise in emphysema. The role of aerobic training has been evaluated in different emphysema models: induced by elastase (Kononov et al., 2001) or papain (Flo et al., 2006). In both models, the aerobic training caused alveolar damage with marked enlargement of distal air spaces. These results were attributed to the stretching of newly deposited elastin and collagen fibers, resulting in larger distortions compared to the normal tissue, thus contributing to emphysema progression (Suki et al., 2005). Otherwise, aerobic training has been shown to induce release of anti-inflammatory mediators (Petersen and Pedersen, 2005; Radom-Aizik et al., 2008; Moir et al., 2010) and reduce levels of growth factors associated with fibrogenesis (O'Callaghan and Williams, 2000; Czarkowska-Paczek et al., 2006; Radom-Aizik et al., 2008; Moir et al., 2010). These differing data may be attributed to: (1) differences in experimental models of emphysema, which do not closely mimic the pulmonary and cardiovascular changes observed in human emphysema; and (2) the timing of exercise initiation, i.e., before, during, or after establishment of emphysema.

We hypothesized that moderate aerobic training, initiated after establishment of emphysema and in animals with recognized cardiorespiratory impairment, would lead to beneficial effects on cardiorespiratory function, as well as modulate proteins implicated in emphysema progression. To answer this question, we investigated the therapeutic effects of aerobic training on lung mechanics, inflammation, remodeling and morphometry, biological markers associated with inflammation, and endothelial cell damage, and cardiac function in a model of elastase-induced emphysema.

## Materials and methods

This study was approved by the Ethics Committee of the Health Sciences Centre, Federal University of Rio de Janeiro (CEUA-CCS-IBCCF 019). All animals received humane care in compliance with the “Principles of Laboratory Animal Care” formulated by the National Society for Medical Research and the “Guide for the Care and Use of Laboratory Animals” prepared by the National Academy of Sciences, USA.

### Animal preparation and experimental protocol

**Figure 1:**
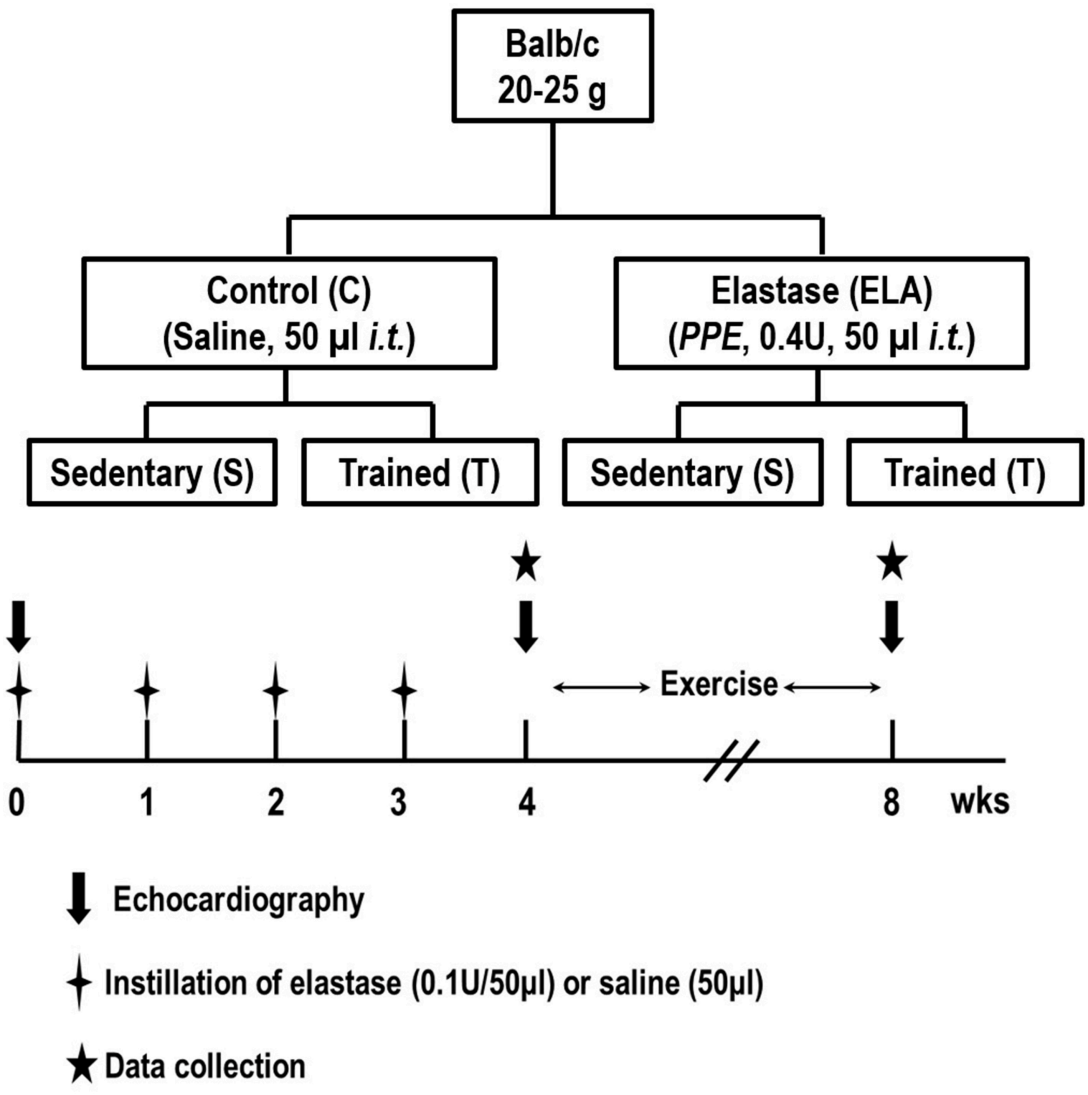
**Schematic flow chart and timeline of study design**. C, control. ELA, elastase-induced emphysema. S, sedentary. T, trained. Data were analyzed at 4 and 8 weeks.

Eighty-four male BALB/c mice (weight 25–30 g) were kept under specific pathogen-free conditions at the animal care facility of the Laboratory of Pulmonary Investigation, Federal University of Rio de Janeiro. Forty-eight mice (*n* = 8/group at 4 and 8 weeks) were used to evaluate *in vivo* lung mechanics and histology, and echocardiographic parameters, while the remaining 36 mice (*n* = 6/group at 4 and 8 weeks) were used to evaluate *in vitro* lung mechanics (Figure 1). Analysis of cytokines and growth factor in lung tissue was performed in all animals. Animals were then randomly assigned using closed sealed envelopes into two groups: control (C) or elastase-induced emphysema (ELA). In the C group, 50μl of sterile saline solution (0.9% NaCl) was intratracheally instilled, while in the ELA group, mice received porcine pancreatic elastase (PPE, 0.1 IU in 50μl of saline solution, Sigma Chemical Co., St. Louis, MO, USA) once weekly for 4 weeks via the same route. For intratracheal instillation, mice were anesthetized with 1.5–2.0% isoflurane by nasal cannula, a midline cervical incision (1 cm) was made to expose the trachea and saline or PPE were instilled using a bent 27-gauge tuberculin needle. The cervical incision was closed with 5–0 silk suture and the mice returned to their cages. One week after the last administration of saline or PPE, mice were further randomized to perform an aerobic training protocol (T) for 4 weeks or no training (sedentary group, S).

### Treadmill aerobic training

Animals from both trained subgroups (C and ELA) were exercised on a motorized treadmill in individualized compartments (EP 132C, Insight, Ribeirão Preto, Brazil). Mice were exercised for 30 min.day^−1^, 3 times/week, at a speed of 8–12 m.min^−1^, 5% grade, during 4 weeks (Lowder et al., 2006), which corresponded to ~65–70% of their maximum oxygen uptake (VO_2max_; Lowder et al., 2005, 2006; Woods et al., 2009). Mice ran without electric shock or prodding. To eliminate the confounding effects of stress due to handling on lung and systemic data, sedentary animals were handled identically to trained ones, including removal from cages the same number of times each day, except during the training session.

### Echocardiography

To evaluate cardiac function in response to pulmonary emphysema and/or aerobic training, echocardiography was performed before saline or PPE instillation (baseline), at the end of the emphysema induction protocol (4 weeks), and at the end of aerobic training protocol (8 weeks). Mice were anesthetized with 1.5–2.0% isoflurane by nasal cannula, shaved over the chest area, and placed in the supine position. Using a 30 MHz mechanical transducer (VEVO 770, Visual Sonics, Toronto, Canada), images were obtained from the short and long axis in B-mode parasternal views. Short-axis parasternal views of the left and the right ventricles were acquired at the level of left papillary muscles to obtain both ventricular areas. Doppler mode was used at the level of the right ventricular outflow tract to assess pulmonary flow. Measurements were obtained in accordance with American Society of Echocardiography Guidelines (Cheitlin et al., 2003; Thibault et al., 2010; Lang et al., 2015).

### Lung mechanics

####  *In vivo*

At 4 and 8 weeks, animals were sedated [diazepam 1 mg intraperitoneally (i.p.)], anesthetized (thiopental sodium 20 mg.kg^−1^ i.p.), tracheotomized, paralyzed (vecuronium bromide, 0.005 mg.kg^−1^ i.v.), and ventilated with a constant-flow ventilator (Samay VR15; Universidad de la Republica, Montevideo, Uruguay) using the following settings: respiratory rate 100 bpm, tidal volume (V_*T*_) 0.2 ml, and fraction of inspired oxygen (Fio_2_) 0.21. The anterior chest wall was surgically removed, and a positive end-expiratory pressure (PEEP) of 2 cmH_2_O was applied. After a 10-min ventilation period, lung mechanics were computed. Airflow and tracheal pressure (Ptr) were measured (Burburan et al., 2007). In an open chest preparation, Ptr reflects transpulmonary pressure (PL). Static lung elastance (Est,L) was computed by the end-inflation occlusion method (Chao et al., 2010). All data were analyzed in the ANADAT software environment (RHT-InfoData, Montreal, Quebec, Canada).

####  *In vitro*

At 4 and 8 weeks, animals were euthanized (thiopental sodium 50 mg.kg^−1^ i.p.), after which lungs were removed *en bloc* and placed in a modified Krebs–Henseleith (K–H) solution (in mM: 118.4 NaCl, 4.7 KCl, 1.2 K_3_PO_4_, 25 NaHCO_3_, 2.5 CaCl_2_·H_2_O, 0.6 MgSO_4_·H_2_O, 11.1 glucose) at pH 7.40 and 6°C (Xisto et al., 2005). Strips of parenchyma (2 × 2 × 10 mm) were cut from the periphery of the left lung and suspended vertically in a K–H organ bath maintained at 37°C, continuously bubbled with a mixture of 95% O_2_/5% CO_2_. Lung strips were weighed (*W*), and their unloaded resting lengths (*L*_0_) were determined with a caliper. Lung strip volume was measured by simple densitometry as vol = Δ*F*/δ, where Δ*F* is the total change in force before and after strip immersion in K–H solution and δ is the mass density of K–H solution. One end of the strip was attached to a force transducer (LETICA TRI-110, Scientific Instruments, Barcelona, Spain), whereas the other was fastened to a lever arm activated by means of a modified woofer driven by the signal generated by a computer and analog-to-digital converted (AT-MIO-16-E-10, National Instruments, Austin, TX, USA). A sidearm of this rod was linked to a second force transducer (LETICA TRI-110, Scientific Instruments, Barcelona, Spain) by means of a silver spring of known Young's modulus, thus allowing measurement of displacement. Neither amplitude dependence (< 0.1% change in stiffness) nor phase changes with frequency were detected in the 0.01–14 Hz range. Cross-sectional unstressed area (*A*_0_) of the strip was determined from volume and unstressed length as *A*_0_ = vol/*L*_0_. Basal force (*F*_B_) for a stress of 0.1 N/cm^2^ was calculated as *F*_B_ (*N*) = 0.1 (*N*/cm^2^). *A*_0_ (cm^2^) and adjusted by vertical displacement of the force transducer as previously described (Romero et al., 2001). The displacement signal was then set to zero. Once basal force and displacement signals were adjusted, the length between bindings (*L*_B_) was measured by means of a precision caliper. Instantaneous length during oscillation around *L*_B_ was determined by adding the value of *L*_*B*_ to the measured value of displacement at any time. Instantaneous average cross-section area (*Ai*) was determined as *Ai* = *Vs*/*Li*(cm^2^). Instantaneous stress (σi) was calculated by dividing force (*g*) by *Ai* (cm^2^). Strain was calculated as Δϵ = (L − L_B_)/L_B_. Each parenchyma strip was preconditioned for 30 min by sinusoidal oscillation of the tissue (frequency = 0.5 Hz; amplitude large enough to reach a final stress of 0.2 N/cm^2^). Thereafter, the amplitude was adjusted to 5% *L*_0_ and the oscillation maintained for another 30 min, or until a stable length-force loop was reached. After preconditioning, the strips were oscillated at a frequency (f) = 1 Hz and tissue elastance (*E*), resistance (*R*), and hysteresivity (η) were calculated (Fredberg and Stamenovic, 1989). Three baseline 20-s recordings were obtained. Both force and displacement signals were preamplified, filtered at 30 Hz (902LPF Frequency Devices, Haverhill, MA, USA), converted to digital (AT-MIO-16E-10, National Instruments Co., Marlboro, MA, USA), and sampled at a frequency of 150 Hz (Software LabVIEW 5.1, National Instruments Co., Austin, TX, USA).

### Lung histology

A laparotomy was performed immediately after determination of *in vivo* lung mechanics, and heparin (1000 IU) was injected into the vena cava. The trachea was clamped at end-expiration, and the abdominal aorta and vena cava were sectioned, yielding a massive hemorrhage that quickly killed the animals.

The left lung was removed, fixed in 4% buffered formalin, and paraffin-embedded. Sections (4 μm thick) were cut and stained with haematoxylin-eosin. Lung morphometry analysis was performed using an integrating eyepiece with a coherent system consisting of a grid with 100 points and 50 lines of known length coupled to a conventional light microscope (Olympus BX51, Olympus Latin America, São Paulo, Brazil). The volume fractions of the lung occupied by collapsed alveoli (alveoli with rough or plicate walls), normal pulmonary areas, or hyperinflated structures (alveolar ducts, alveolar sacs, or alveoli, all with maximal chord length in air >120 μm) were determined by the point-counting technique (Cruz-Orive and Weibel, 1990) across 10 random, non-coincident microscopic fields. Briefly, points falling on collapsed, normal pulmonary areas, or hyperinflated structures were counted and divided by the total number of points in each microscopic field. Enlargement of air spaces was evaluated using mean linear intercept measurement (Lm) on whole lung at 400 × magnification. Number of neutrophils and mononuclear (MN) cells and lung tissue were evaluated at 1000 × magnification. Points falling on neutrophils and MN cells were counted and divided by the total number of points falling on lung tissue in each microscopic field. Collagen (Picrosirius-polarization method) and elastic fibers (Weigert's resorcin-fuchsin method with oxidation) were quantified in the pulmonary parenchyma (Chao et al., 2010), with the aid of a digital analysis system and specific software (Image-Pro Plus 7.0 for Windows; Media Cybernetics, Silver Spring, MD), under 200 × magnification. The images were generated by a microscope (Axioplan, Zeiss) connected to a camera (Sony Trinitron CCD, Sony, Tokyo, Japan), fed into a computer through a frame grabber (Oculus TCX, Coreco, St Laurent, QC, Canada) for offline processing. The thresholds for collagen and elastic fibers were established after enhancement of contrast up to the point where the fiber was easily identified as either birefringent (collagen) or black (elastic) bands. Bronchi and blood vessels were carefully avoided during the measurements. To avoid any bias due to septal edema or alveolar collapse, the areas occupied by the elastic and collagen fibers were measured by digital densitometric recognition, divided by the tissue of each studied area, and expressed as the percentage of elastic and collagen fibers in pulmonary parenchyma.

### Transmission electron microscopy

Three sections (2 × 2 × 2 mm) were cut from three random and different segments of the right lung and fixed in 2.5% glutaraldehyde and phosphate buffer 0.1 M (pH = 7.4) for electron microscopy analysis (JEOL 1010 Transmission Electron Microscope, Tokyo, Japan). In each electron microscopy image (*n* = 20/animal), the following parameters were analyzed: abnormal enlargement of airspaces with thin and irregular alveolar walls, alveolar-capillary membrane damage, collagen fiber deposition, type 2 epithelial cell damage, and endothelial cell damage. Pathologic findings were graded on a five-point, semi-quantitative, severity-based scoring system as follows: 0 = normal lung parenchyma, 1 = changes in 1–25% of examined tissue, 2 = changes in 26–50% of examined tissue, 3 = changes in 51–75% of examined tissue, and 4 = changes in 76–100% of examined tissue (de Araujo et al., 2012).

Lung histological analysis (light and electron microscopy) were performed in a blinded fashion by the same pathologist.

### Lung cell apoptosis

Terminal deoxynucleotidyl transferase biotin-dUTP nick end labeling (TUNEL) staining was performed to evaluate cell apoptosis (de Araujo et al., 2012). Twenty fields per section from regions with cell apoptosis were examined at 400 × magnification. Results were expressed as percentage of apoptotic cells in lung parenchyma.

### Enzyme-linked immunosorbent assay (ELISA)

Levels of interleukin (IL)-1β, tumor necrosis factor (TNF)-α and vascular endothelial growth factor (VEGF) in lung tissue was evaluated by ELISA, performed in accordance with the manufacturer's instructions (Duo Set, R&D Systems, Minneapolis, MN, USA).

### Statistical analysis

Data were tested for normality of distribution (Kolmogorov-Smirnov test with Lilliefors' correction) and homogeneity of variances (Levene's median test). At 4 weeks, the C and ELA groups were compared using Student's *t*-test or the Mann–Whitney *U* test as appropriate. At 8 weeks, in order to test whether moderate aerobic training, would lead to beneficial effects on cardiorespiratory function, two-way analysis of variance (ANOVA) followed by Tukey's or Kruskal–Wallis test was used. One-way ANOVA for repeated measures followed by Tukey's test was used to compare echocardiographic data along time. Parametric data were expressed as mean ± standard deviation, whereas nonparametric data were expressed as median (interquartile range). All tests were performed using the SPSS for Windows version 18.0 (SPSS, Chicago, IL), and statistical significance was established as *P* < 0.05.

## Results

**Figure 2:**
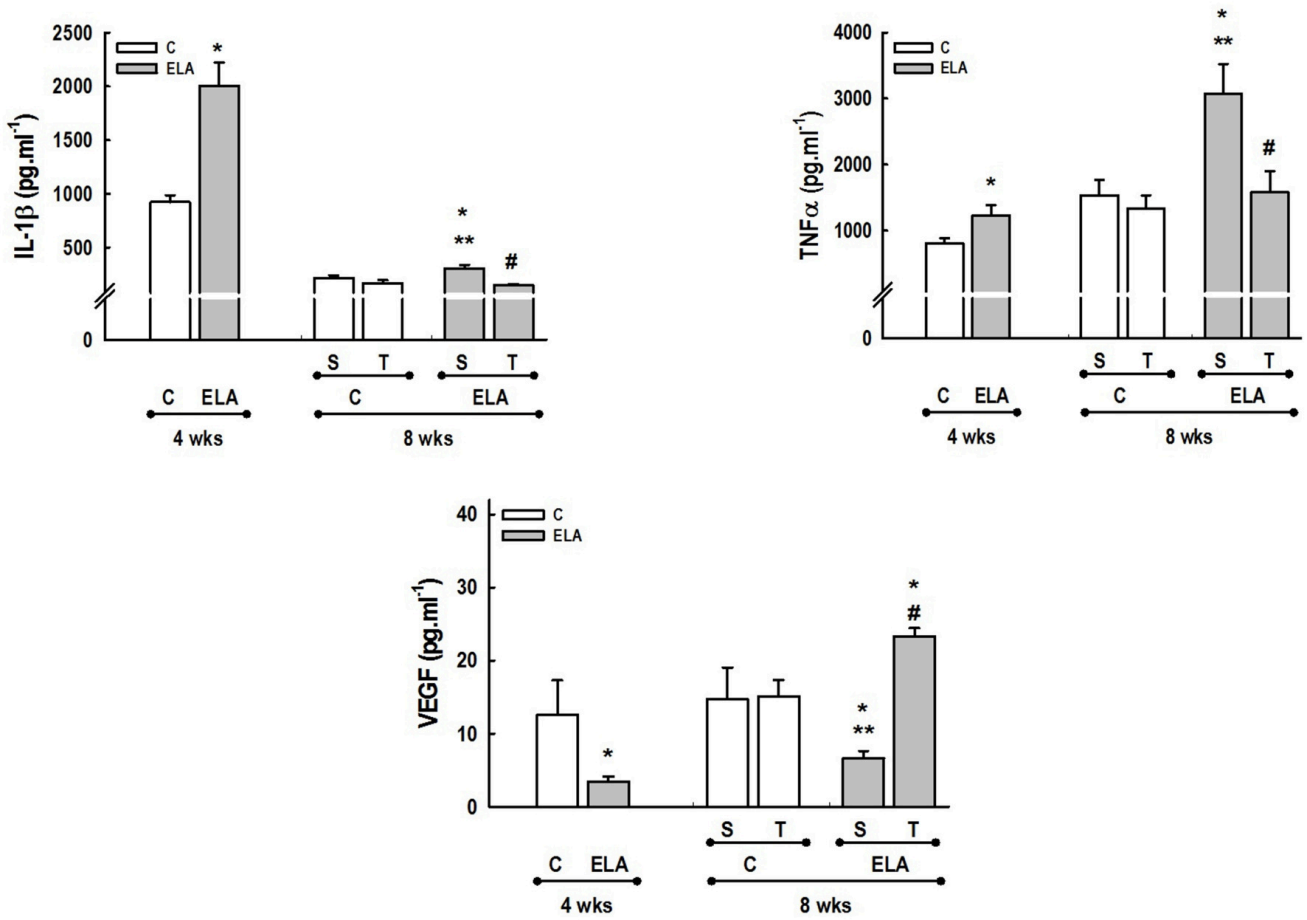
**Levels of interleukin (IL)-1β, tumor necrosis factor (TNF)-α, and vascular endothelial growth factor (VEGF) in lung tissue**. C, control. ELA, elastase-induced emphysema. S, sedentary. T, trained. Values are means (±*SD*) of eight animals in each group. ^*^Significantly different from C group at 4 weeks (*p* < 0.05). ^**^Significantly different from respective C group at 8 weeks (*p* < 0.05). ^#^Significantly different from ELA-S group (*p* < 0.05).

**Figure 3:**
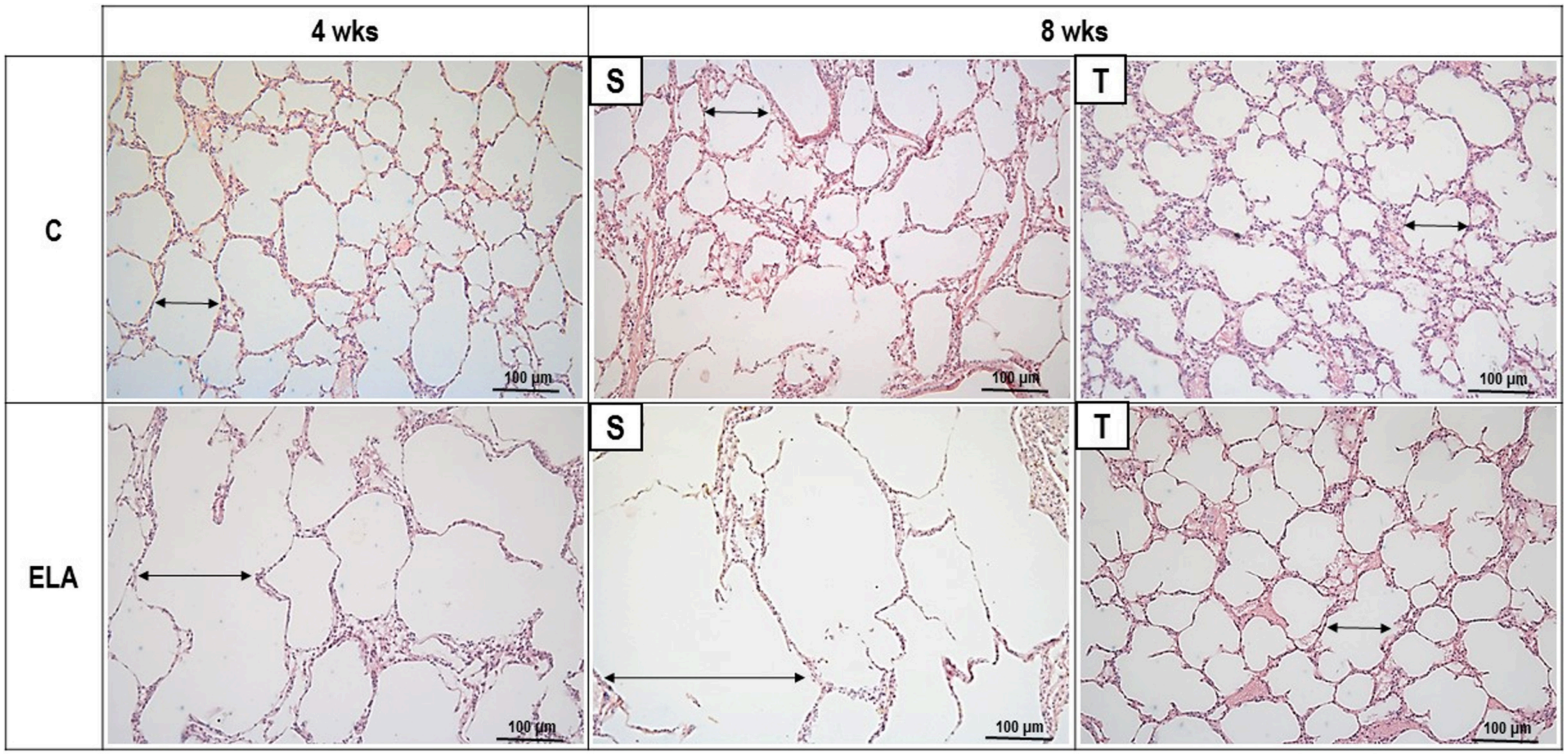
**Representative photomicrographs of lung parenchyma stained with hematoxylin–eosin (H&E)**. C, control. ELA, elastase-induced emphysema. S, sedentary. T, trained. Double arrows, alveolar diameter.

**Figure 4:**
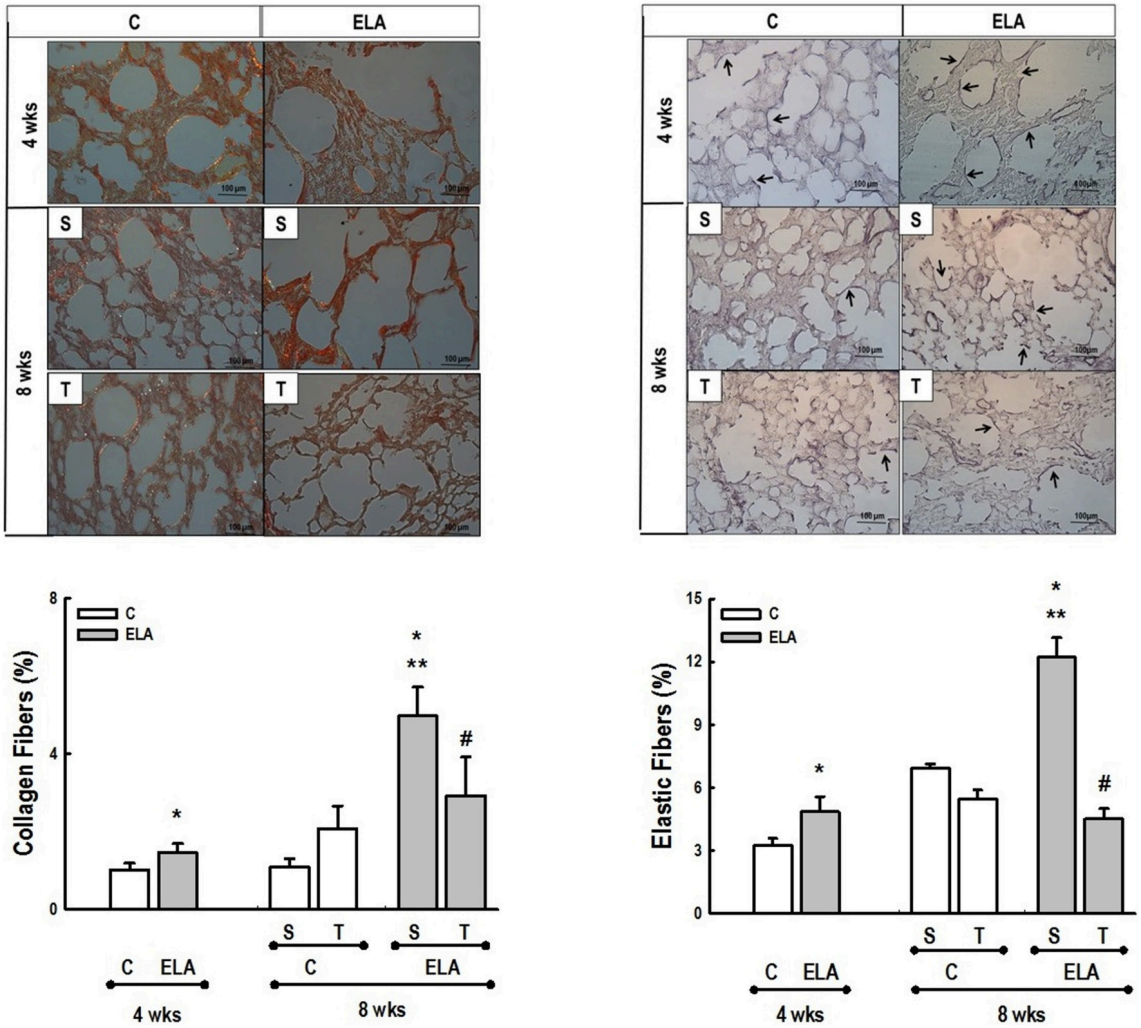
**Representative photomicrographs of lung parenchyma stained with Picrosirius-polarization method (collagen fibers) and Weigert's resorcin fuchsin method with oxidation (elastic fibers)**. C, control. ELA, elastase-induced emphysema. S, sedentary. T, trained. Arrows, Elastic fibers (stained black). Values are means (±*SD*) of eight animals in each group. Data were gathered from 10 random, non-coincident fields per mouse. ^*^Significantly different from C group at 4 weeks (*p* < 0.05). ^**^Significantly different from respective C group at 8 weeks (*p* < 0.05). ^#^Significantly different from ELA-S group (*p* < 0.05).

**Figure 5:**
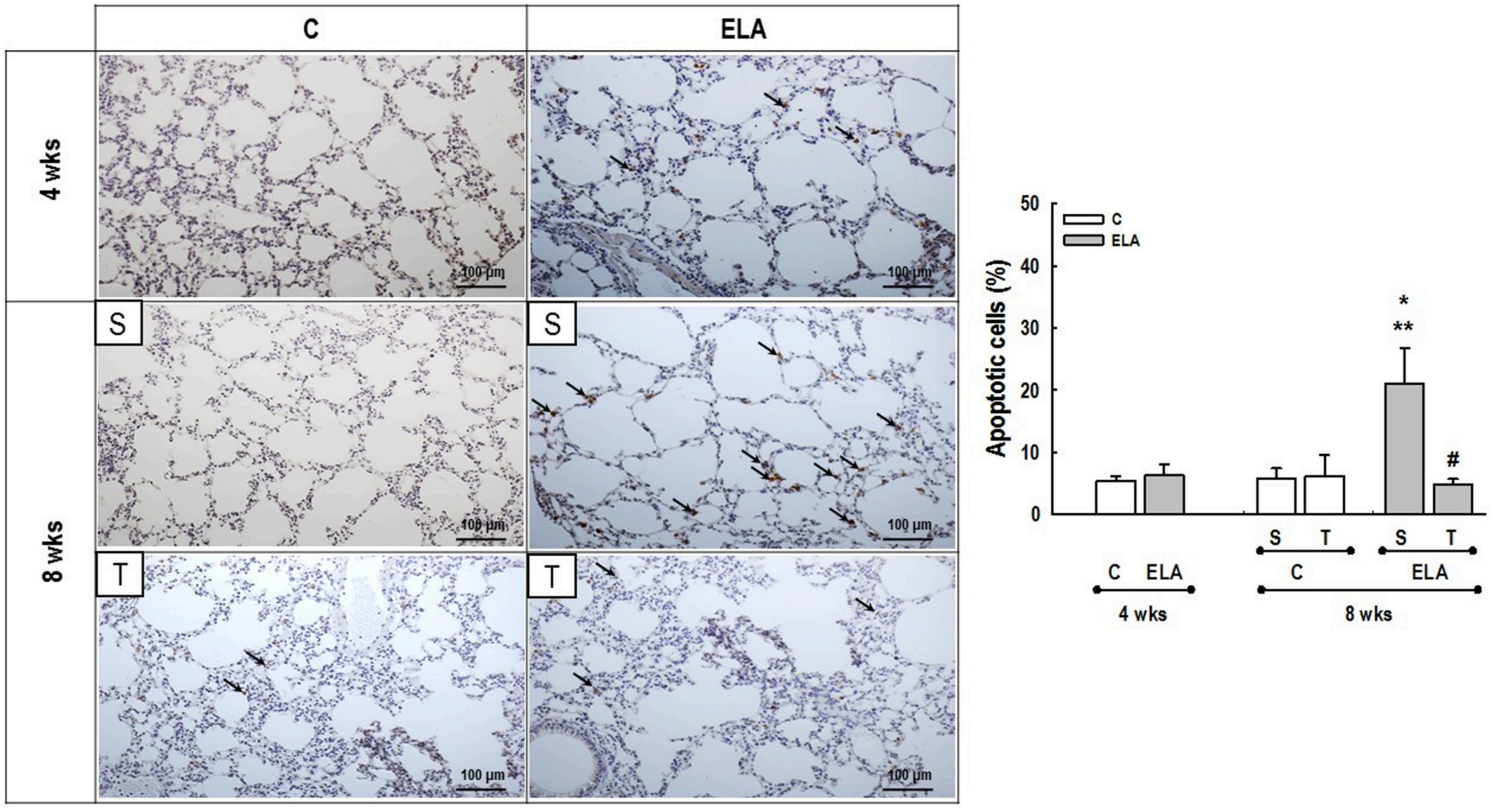
**Representative photomicrographs of lung parenchyma stained with TUNEL technique to detect apoptotic cells**. C, control. ELA, elastase-induced emphysema. S, sedentary. T, trained. Arrows, brown-stained TUNEL-positive cells. Values are means (±*SD*) of eight animals in each group. Data were gathered from 20 random, non-coincident fields per mouse. ^*^Significantly different from C group at 4 weeks (*p* < 0.05). ^**^Significantly different from respective C group at 8 weeks (*p* < 0.05). ^#^Significantly different from ELA-S group (*p* < 0.05).

**Figure 6:**
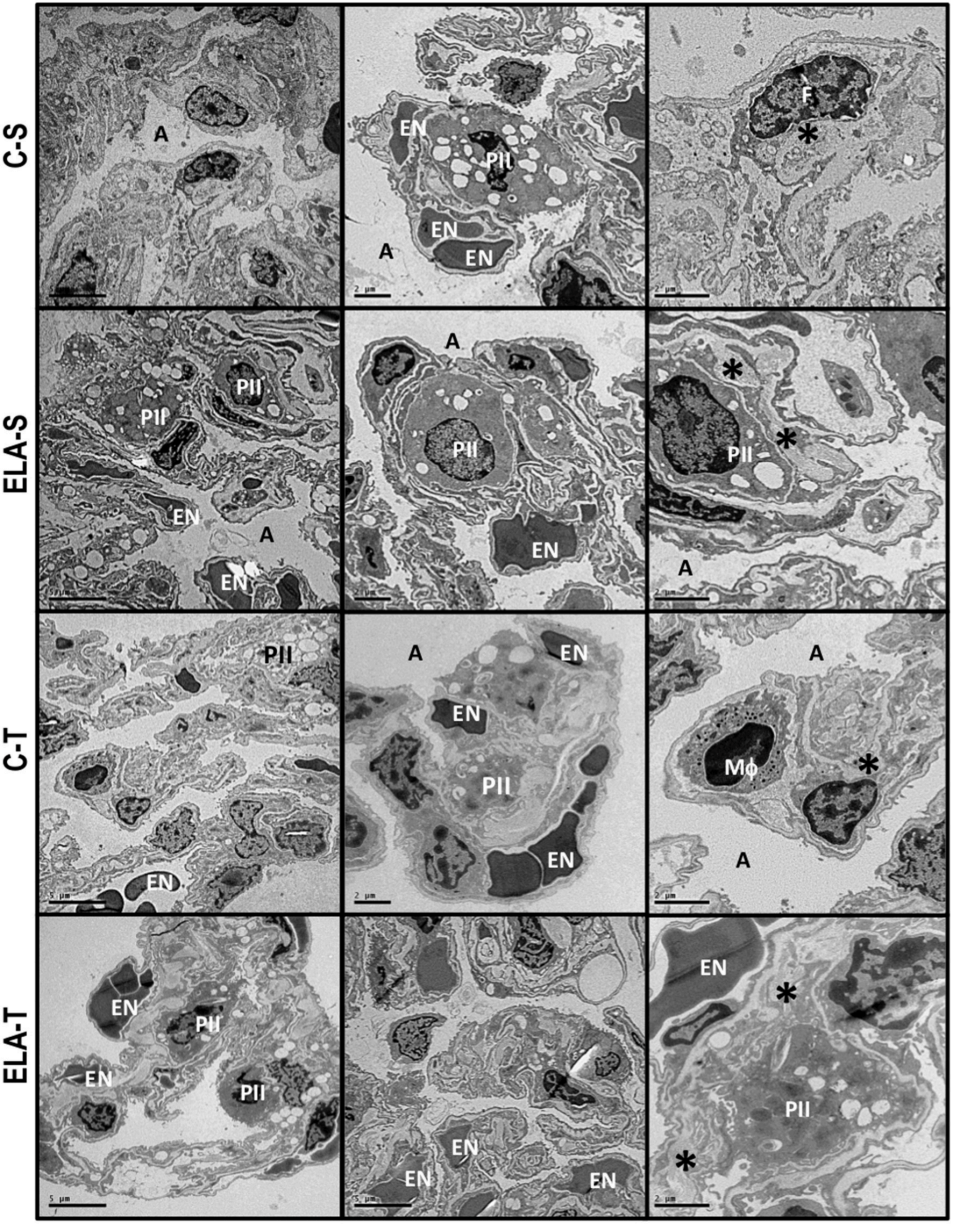
**Transmission electron microscopy of alveolar architecture 4 weeks after the last instillation of saline (C) or elastase (ELA) instillation in sedentary (S) and trained (T) mice**. Photomicrographs are representative of data obtained from lung sections of eight animals in each group. Both C-S and C-T present normal alveolar space (A), preserved alveolar-capillary membrane and endothelium (EN), type II pneumocytes (PII), and interstitial fibroblasts (F), as well as collagen fiber (^*^). Note the rupture of alveolar septa (AS) associated with injured endothelial cells, decreased number of capillaries and lamellar bodies in type II pneumocytes (PII), and increased number of fibroblasts (F) immersed in increased collagen fibers (^*^) in ELA-S. In ELA-T, alveolar-capillary membrane was less damaged and collagen fiber content reduced.

**Figure 7:**
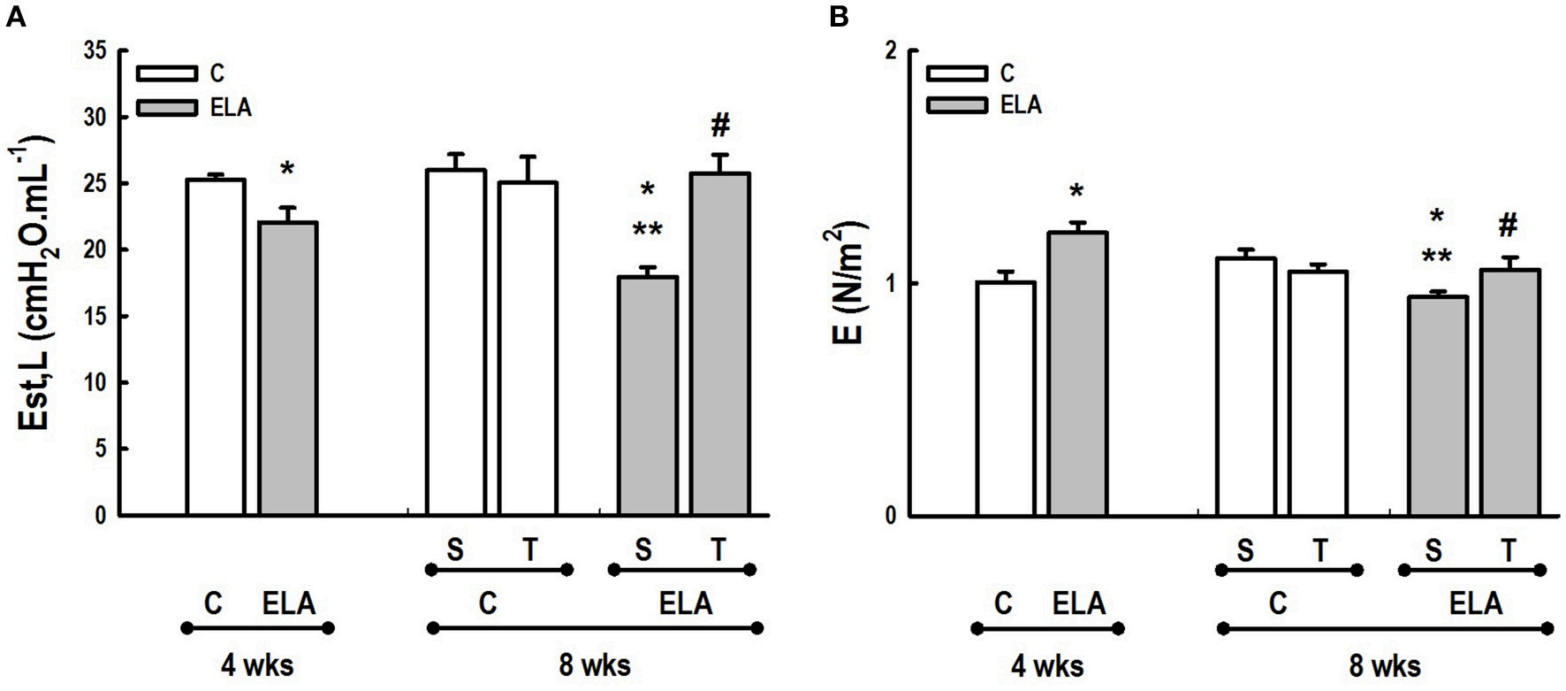
**(A)** *In vivo* lung mechanics. Est,L, Static lung elastance. **(B)** *In vitro* lung mechanics. E, elastance. C, control. ELA, elastase-induced emphysema. S, sedentary. T, trained. Values are means (±*SD*) of eight animals in each group. ^*^Significantly different from C group at 4 weeks (*p* < 0.05). ^**^Significantly different from respective C group at 8 weeks (*p* < 0.05). ^#^Significantly different from ELA-S group (*p* < 0.05).

**Figure 8:**
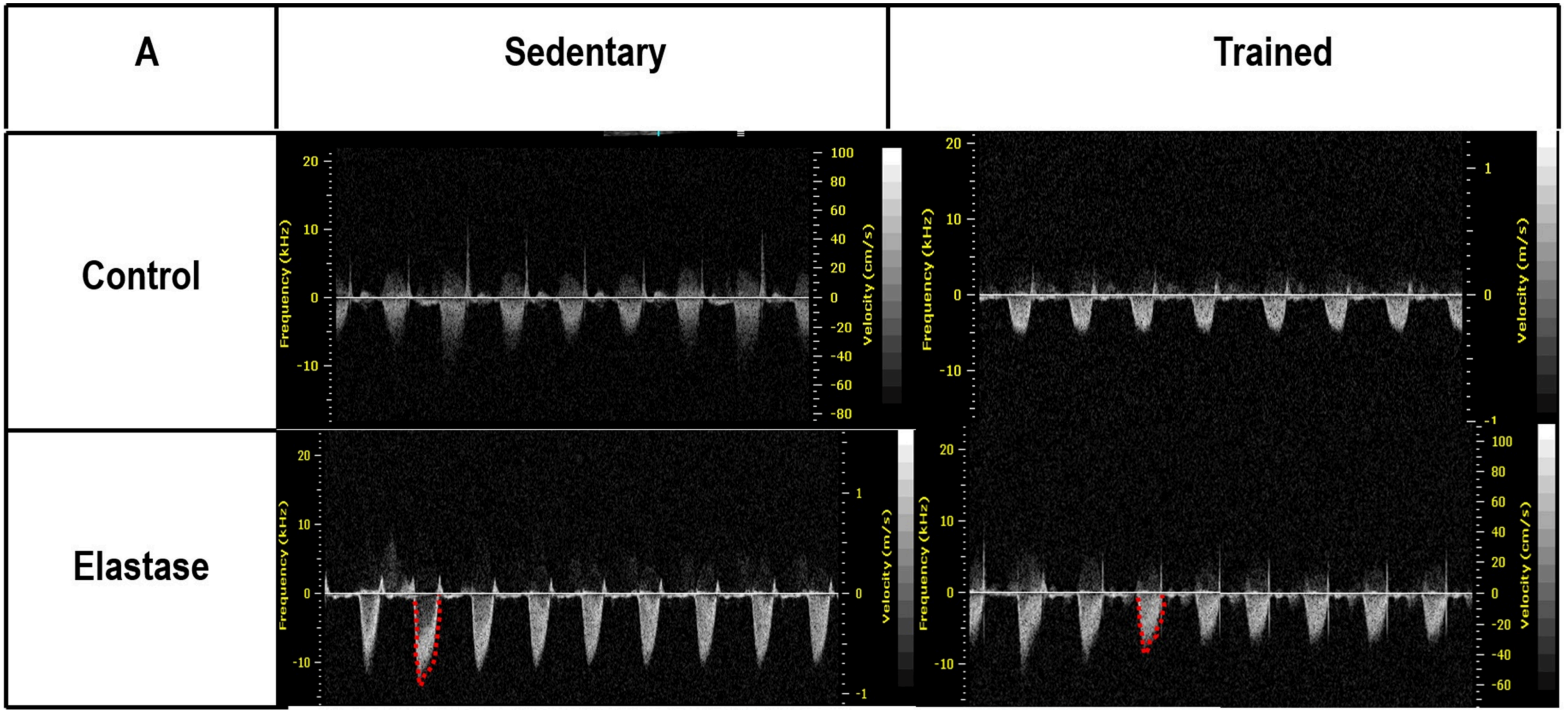
**Echocardiographic parameters**. Pulmonary artery Doppler flow pattern. C, control. ELA, elastase-induced emphysema. S, sedentary. T, trained.

After 4 weeks of emphysema induction, ELA compared to C group exhibited: (1) higher protein levels of IL-1β and TNF-α, and decreased of VEGF (Figure 2); (2) increased alveolar collapse, lung hyperinflation, mean linear intercept, and number of neutrophils (Table 1, Figure 3); (3) increased collagen and elastic fiber content (Figure 4) and number of apoptotic cells in lung tissue (Figure 5); (4) ultrastructural damage of epithelial and endothelial cells, elastolysis, and increased number of fibroblasts (Table 2, Figure 6); (5) decreased static lung elastance (Est,L; *in vivo* lung mechanics) and increased tissue elastance (Figure 7), with no changes in tissue resistance or hysterisivity (*in vitro* lung mechanics; data not shown); and 6) increased right ventricular diastolic area (RVA; Table 3, Figure 8). No alterations were observed in left ventricular area (data not shown).

**Table 1:** **Lung Morphometry**.

		**Normal (%)**	**Collapse (%)**	**Hyperinflation (%)**	**Lm (μm)**	**Neutrophils (%)**	**MN cells (%)**	**Total cells (%)**
**4 WEEKS**								
C		97.0 ± 2.4	1.8 ± 2.4	1.2 ± 1.2	48.0 ± 5.2	2.8 ± 1.3	26.0 ± 5.2	28.8 ± 4.9
ELA		80.5 ± 10.2^*^	2.9 ± 2.6	16.6 ± 10.3^*^	62.5 ± 8.9^*^	4.3 ± 1.5^*^	27.3 ± 3.7	31.6 ± 3.4
**8 WEEKS**								
C	S	96.8 ± 2.6	3.2 ± 2.6	0.0 ± 0.0	46.4 ± 10.0	0.5 ± 0.5	24.7 ± 6.5	34.3 ± 3.3
	T	98.2 ± 2.3	1.8 ± 2.0	0.0 ± 0.0	45.5 ± 5.8	2.5 ± 1.8	30.3 ± 5.3	33.1 ± 6.0
ELA	S	54.1 ± 7.6 ^**^	16.9 ± 9.9^**^	28.9 ± 16.4^**^	82.2 ± 30.9 ^**^	4.9 ± 1.9^**^	28.5 ± 3.2	33.4 ± 3.1
	T	78.9 ± 5.5^**^^,^ ^#^	14.9 ± 6.3^**^	6.2 ± 3.1^#^	47.7 ± 4.0^#^	6.4 ± 1.4 ^**^^,^ ^#^	26.0 ± 3.7	32.3 ± 3.9

Values are means (±SD) of eight animals in each group. Fractional area of normal, collapsed, and hyperinflated alveoli. Lm, mean linear intercept.

Percentage of neutrophil, mononuclear (MN), and total cells. All values were computed in 10 random, non-coincident fields of view per mouse. C, control. ELA, elastase-induced emphysema. S, sedentary. T, trained.

* Significantly different from C group at 4 weeks (p < 0.05).

** Significantly different from respective C group at 8 weeks (p < 0.05).

# Significantly different from ELA-S group (p < 0.05).

**Table 2:** **Semiquantitative analysis of electron microscopy**.

		**Alveolar-capillary damage**	**Type 2 epithelial cells**	**Collagen fiber deposition**	**Increased number of fibroblasts**	**Rupture of alveolar walls**
**4 WEEKS**						
C		0 (0–0.5)	0 (0–0)	0 (0–0.5)	0 (0–0.5)	0 (0–0)
ELA		3 (2–3)^*^	3 (2–3)^*^	2 (1.5–2)^*^	3 (2.5–3.5)^*^	2 (2–2.5)^*^
**8 WEEKS**						
C	S	0 (0–0)	0 (0–0)	0 (0–0.5)	0 (0–0)	0 (0–0)
	T	0 (0–0.5)	0 (0–0)	0 (0–0)	0 (0–0.5)	0 (0–0)
ELA	S	3 (2.5–4)^**^	3 (2–3.5)^**^	4 (3–4)^**^	3 (2.5–4)^**^	3 (3–4)^**^
	T	2 (2–3)^**^	2 (1.5–2)^**^	2 (1–2)^**^^,^ ^#^	2 (1.5–3)^**^	2 (1.5–2)^**^^,^ ^#^

Values are median (interquartile range) of five animals per group. Pathologic findings were graded on a five-point, semiquantitative, severity-based scoring system: 0 = normal lung parenchyma, 1 = changes in 1–25% of examined tissue, 2 = 26 to 50% of examined tissue, 3 = 51 to 75% of examined tissue, and 4 = 76 to 100% of examined tissue. C, control. ELA, elastase-induced emphysema. S, sedentary. T, trained.

* Significantly different from C group at 4 weeks (p < 0.05).

** Significantly different from respective C group at 8 weeks (p < 0.05).

# Significantly different from ELA-S group (p < 0.05).

**Table 3:** **Echocardiographic data**.

	**Weeks**	**C**		**ELA**	
		**S**	**T**	**S**	**T**
Right ventricular diastolic area (mm2)	0	11.3 ± 1.0	11.3 ± 0.9	11.6 ± 0.5	11.9 ± 0.8
	4	11.7 ± 1.2	12.0 ± 1.1	15.6 ± 0.4^*^	15.5 ± 0.4^*^
	8	13.2 ± 0.4	13.5 ± 0.8	16.0 ± 0.3^*^	13.7 ± 1.3^#^

Values are means (±SD) of eight animals in each group. C, control. ELA, elastase-induced emphysema. S, sedentary. T, trained.

* Significantly different from respective C group.

# Significantly different from ELA-S group (p < 0.05).

Aerobic training did not affect any of the aforementioned parameters in the C groups. Regarding the ELA subgroups, trained (T) compared to sedentary (S) animals exhibited: (1) an increased number of neutrophils in lung tissue (Table 1, Figure 3); (2) reduced alveolar hyperinflation, mean linear intercept (Table 1), and number of apoptotic cells in lung tissue (Figure 5); (3) decreased collagen and elastic fiber content (Figure 4), elastolysis, and alveolar wall rupture (Table 2, Figure 6); (4) reduced levels of TNF-α (Figure 2); (5) increased VEGF level (Figure 2), Est,L and E (Figure 7). Moreover, aerobic training reduced RVA (Table 3, Figure 8). No differences in left ventricular area were found among groups (data not shown).

## Discussion

In this study, mice were subjected to a protocol of multiple doses of elastase and, once identified the cardiorespiratory function impairment associated with emphysema (4 weeks after induction), aerobic training was then initiated and performed for 4 weeks. This is the first experimental study using an emphysema model with repetitive elastase instillations protocol to evaluate the therapeutic effect of moderate aerobic training. Exercise protocol resulted in decreased mean linear intercept, lung hyperinflation, number of apoptotic cells, collagen deposition and rupture of alveolar walls, levels of TNF-α in lung tissue, as well as increased VEGF levels, static lung elastance (*in vivo*), and tissue elastance (*in vitro*). Moreover, aerobic training reduced RVA. Therefore, in agreement with our hypothesis, in this translational study setting, moderate aerobic training produced effects desirable in an adjunctive therapeutic strategy for emphysema, modulating the pulmonary inflammatory and remodeling processes induced by emphysema and reducing pulmonary arterial hypertension.

Several experimental models have been used in an attempt to reproduce the characteristics of human emphysema. The most widely used are inhalation of cigarette smoke (Sarir et al., 2010; Chen et al., 2011; Barreiro et al., 2012; Toledo et al., 2012) and instillation of elastolytic enzymes (PPE, papain or human neutrophil elastase) (Cheng et al., 2009; Inoue et al., 2010; Mador et al., 2010; Zhang et al., 2010; Ishii et al., 2012). In this study, we used the model of multiple instillations of PPE previously described by Luthje et al. (2009) and modified by our group (Cruz et al., 2012; Padilha et al., 2015), which has been demonstrated to yield the lung and cardiovascular damage. Additionally, this model yields lengthy progressive inflammatory and remodeling processes, as well as alveolar destruction and worse lung function in the time course of the disease.

Aerobic training was performed on a motorized treadmill for 30 min, at a speed of 8–12 m.min^−1^ and 5% grade, corresponding to ~65–70% of maximal oxygen uptake (VO_2max_), as determined by previous studies (Lowder et al., 2005, 2006). The exercise protocol was applied for 4 weeks, and this mode of exercise allows better monitoring of intensity and duration as compared with swimming or voluntary running (Pastva et al., 2004).

Emphysema is characterized by alveolar extracellular matrix destruction, resulting in airspace enlargement and reduction in alveolar-capillary exchange area (Morris and Sheppard, 2006; Hantos et al., 2008). In this study, we observed a time dependence of lung mechanics and histology. At 4 weeks, the reduction in Est,L reflected the decrease in lung elastic recoil, which is consistent with the increase in mean linear intercept (Lopes et al., 2009, 2013; Anciaes et al., 2011). This could be explained by two factors: (1) the emphysema working values were in the non-steep part of the respiratory system PV curve due to tissue loss; (2) the decrease in lung elastance is in line with great collagen and elastic fibers depositions. It is well-known that the increase in mean linear intercept cannot fully explain the decrease in matrix breakdown, especially when the collagen content per alveolar wall is increased (Ito et al., 2005). To test this hypothesis, we confirmed that the increase in tissue elastance was associated with increased deposition of collagen and elastic fibers (Figures 4, 7). At 8 weeks, deposition of elastic and collagen fibers was even greater compared to 4 weeks, but tissue elastance was lower in ELA-S compared to C-S animals. These findings run counter to those observed in fibrotic lungs, in which total collagen content also increased, but was accompanied by an increase in stiffness (Dolhnikoff et al., 1999). We may infer that the remodeling process—more precisely, the internal structural organization of the collagen fibers in the alveolar wall—should be different in emphysematous and fibrotic lungs (Suki et al., 2005). Some reports have shown a disorganized collagen deposition in emphysema, which the mechanical forces can break the alveolar walls during breathing process (Suki et al., 2003; Martin-Mosquero et al., 2006), contributing to progressive damage. TNF-α also increased from ELA at 4 weeks to ELA-S at 8 weeks (Figure 2), depicting a time dependence of this emphysema model, similar to that described in an *in vitro* model (Demirjian et al., 2006). The raised levels of TNF-α can be associated to higher macrophage phenotype 1 (M1), which has pro-inflammatory features, instead to regenerative macrophage phenotype 2 (M2; Padilha et al., 2015). Furthermore, the increase in TNF-α levels along time was associated with the increased number of apoptotic cells (Figure 5), which shows a disease progression in this emphysema model.

It has been demonstrated that, in the emphysematous lung, alveolar walls can break at physiological stretch corresponding to normal breathing, suggesting that newly synthetized collagen could be weaker (Suki et al., 2005). Although we did not measure the dynamics of breathing in mice during aerobic training, we may infer that alveolar stretch might increase, which could be an important mechanical force acting toward alveolar wall breakdown (Mishima et al., 1999). However, we observed that, after moderate aerobic training, mean linear intercept, collagen, and elastic fiber content were reduced compared to those of sedentary animals. This was followed by an increase in Est,L and E. We may hypothesize that aerobic training does not generate a lung stretch strong enough to affect collagen and elastic fibers so as to stimulate matrix turnover. In contrast, it positively modulated the remodeling process, leading to elastic recoil restoration. In a previous study, Flo et al. demonstrated that exercise training starting 2 days after intratracheal papain infusion increased the severity of alveolar damage, worsening emphysema (Flo et al., 2006). The dissociation between the previous study and our results could be explained by: (1) the different agents (papain vs. elastase) used to induce emphysema, (2) the degree of exercise (6 days/week for 9 weeks vs. 3 days/week for 4 weeks), and (3) the interaction between acute papain exposure and intense exercise sessions. Taking into account the limitations related to extrapolation of animal experimentation findings to human conditions, it should be noted that rehabilitation starts when cardiorespiratory impairment is already present in patients with emphysema. In the present study, we observed signs of pulmonary hypertension after 4 and 8 weeks, characterized by increased RVA (Koskenvuo et al., 2010). Moderate aerobic exercise minimized the cardiovascular alterations observed in the ELA-S group (Figure 8). In line with these results, VEGF levels increased in response to moderate exercise training (Figure 2). This increase in VEGF level is consistent with attenuation of air space enlargement (Takahashi et al., 2013) and exercise-induced angiogenesis, which may contribute to increased vascular cross-section area and reduced right ventricular overload (Gustafsson and Kraus, 2001; da Silva et al., 2012).

Regular and moderate exercise improves the immune response by reducing production of inflammatory cytokines (Lee et al., 2010; Gholamnezhad et al., 2014). Moderate exercise training in mice exposed to cigarette smoke minimized the expression of monocyte chemoattractant protein-1 (MCP-1) by inflammatory cells in the lung parenchyma (Toledo et al., 2012). Furthermore, previous studies have shown that aerobic training in allergic lung inflammation (Vieira et al., 2007) and sepsis (Chen et al., 2007) decreased lung inflammation. In this line, we observed that aerobic training reduced levels of IL-1β and TNF-α in lung tissue, suggesting a reduction in local inflammatory response (Mooren et al., 2012; Weng et al., 2013). Not only lung inflammation, but also the percentage of lung cell apoptosis reduced after aerobic training. Aerobic exercise was associated with a delay in neutrophil apoptosis, indicating alert and activation of the nonadaptive immune system (Mooren et al., 2012). The consequence of this decrease in apoptosis rate would be an increase of peripheral neutrophil numbers, which was indeed observed in the present study (Table 1). During and after exercise, catecholamines and cortisol are released, and these substances may facilitate the transition of neutrophils from endothelial walls and bone marrow (Korchak et al., 1998). Although, ELA-T showed an increase in tissue neutrophils percentage, no further structural alterations were observed in this group due to their action, as elastolysis, which by the way, decreased. Furthermore, it should be noted that delays in apoptosis are observed in various inflammatory conditions (Maianski et al., 2004), and it may be related to neutrophils staying in lung tissue.

### Limitations

The present study has several limitations that need to be addressed. First, although intratracheal administration of elastase has been considered a good model of pulmonary emphysema (Antunes and Rocco, 2011), whether these results could be translated to the human condition is still unclear. Second, we only measured inflammatory mediators in lung tissue; thus, we did not assess the systemic effects of aerobic exercise in this model. This could be done by measuring similar mediators, but in mice, the amount of blood available for sampling is usually less than that required for such measurements. Third, we did not measure the VO_2_ of the animals during exercise. However, according to previous studies (Lowder et al., 2005, 2006), the training protocol used herein corresponds to ~65–70% of VO_2max_. This range is slightly higher to that advocated by the Global initiative for chronic Obstructive Lung Disease, which is 50% (Decramer et al., 2015).

## Conclusion

Regular and moderate therapeutic exercise in elastase-induced emphysema modulated lung inflammation and remodeling, and reduced pulmonary arterial hypertension. This study provides evidence that moderate aerobic exercise had beneficial effects on lung morphofunctional parameters and inflammatory mediators, as well as on cardiovascular function, in an animal model of emphysema.

## Author contributions

Conceived and designed the experiments: IH, ML, GP, NR, PS, DX, PR; Performed experiments: IH, ML, GP, PM, RM, MA, NR, DX; Analyzed data: IH, ML, GP, PM, RM, MA, MM, NR, PS, DX, PR; Interpreted results of research: IH, ML, GP, PM, RM, MA, MM, NR, PS, DX, PR; Drafted, edited, critically revised paper: IH, ML, PS, PR; All authors read and approved final version of manuscript.

## Funding

This study was supported by the Brazilian Council for Scientific and Technological Development (CNPq), the Rio de Janeiro State. Research Foundation (FAPERJ), the Department of Science and Technology (DECIT)/Brazilian Ministry of Health, and the Coordination for the Improvement of Higher Education Personnel (CAPES).

### Conflict of interest statement

The authors declare that the research was conducted in the absence of any commercial or financial relationships that could be construed as a potential conflict of interest. The reviewer TL and handling Editor declared their shared affiliation, and the handling Editor states that the process nevertheless met the standards of a fair and objective review.
